# Identifying Role Functions of Primary Health Care Nurses in China: A Mixed‐Methods Study in Three Northeastern Provinces

**DOI:** 10.1155/jonm/3708836

**Published:** 2026-03-03

**Authors:** Jiashuo Zhang, Wenna Qian, Jialin Li, Mengyao Cui

**Affiliations:** ^1^ School of Nursing, China Medical University, Shenyang, China, cmu.edu.tw; ^2^ Department of Nursing, The Second Hospital of Dalian Medical University, Dalian, China, dlmedu.edu.cn; ^3^ Department of Breast Surgery, The First Hospital of China Medical University, Shenyang, China, cmu.edu.cn

**Keywords:** nurses, primary health care, role functions, tasks

## Abstract

**Background:**

With global aging and the increasing burden of noncommunicable diseases (NCDs), the primary health care (PHC) system plays a pivotal role in disease prevention and management of NCDs. Nurses, as key PHC members, significantly influence health equity and service quality.

**Objective:**

To examine tasks and role functions of Chinese PHC nurses.

**Methods:**

A mixed‐methods convergent study was conducted and reported in accordance with the Good Reporting of a Mixed Methods Study (GRAMMS) framework. A cross‐sectional survey and focus group interviews with nurses from 10 PHC institutions in Northeast China were conducted. The Chinese Nurses’ Task Survey Questionnaire was used to collect data on the tasks of nurses. Face‐to‐face semistructured focus group interviews were conducted to explore the nurses’ perceptions, understandings, and attitudes toward their tasks. Quantitative data were collated and analyzed using EpiData 3.1 and SPSS 27.0 software, and qualitative data were analyzed through natural language processing (NLP)–based BERTopic topic modeling and conventional content analysis. The quantitative and qualitative results were integrated, merged, and refined to construct the role functions of PHC nurses in China.

**Results:**

A total of 123 PHC nurses (*n* = 123) completed the survey, and 10 focus group interviews were conducted with 75 participants (*n* = 75). The results indicated that the current tasks of PHC nurses in China were mainly focused on providing basic medical services, health management, and health education. Three core role functions of Chinese PHC nurses were summarized and refined: guardian of family health, early warner of major diseases, and facilitator of health promotion.

**Conclusions:**

Compared with previous studies, the scope of role functions of PHC nurses in China has expanded, with a greater emphasis focus on the management of common NCDs among residents. However, the roles of PHC nurses could not be fully fulfilled, and functional deficiencies still existed.

**Implications for Nursing Management:**

Healthcare managers should fundamentally clarify the role positioning and job responsibilities of PHC nurses. Relevant policies and regulations should be formulated, standardized training systems established, financial compensation mechanisms provided, and professional title promotion pathways optimized to maximize the potential of PHC nurses, thereby enhancing the accessibility of medical services.

## 1. Introduction

Universal health coverage (UHC) [1] aims to ensure that all individuals can access the full range of high‐quality health services they need when and where required, without facing financial hardship. This encompasses a comprehensive range of basic medical and health services across the life course, from health promotion and disease prevention to treatment, rehabilitation, and palliative care. Achieving universal coverage of basic health services is a critical requirement outlined in the “Healthy China 2030” blueprint [2]. However, over the past decade, China has witnessed a sustained increase in the prevalence and disease burden of noncommunicable diseases (NCDs) [3], which poses a substantial challenge to the achievement of UHC [4]. At the same time, inequality remains a fundamental obstacle to achieving UHC [5–7]. Primary health care (PHC) is a vital strategy for addressing these issues [4, 6, 7], and the Chinese government has implemented a series of policy measures to strengthen the PHC system, focusing on preventing and controlling NCDs [8], managing common health problems, and responding to public health emergencies. The number of PHC nurses in China has doubled, reaching 1.15 million, accounting for one‐fifth of the national total nursing workforce [9]. As community residents’ health literacy continues to improve and demand more diverse health needs, defining the roles of PHC nurses is essential to enable them to serve the health needs of community residents better [10], directly impacting health equity and the quality patients received [11, 12]. Despite the strengthening of the PHC system, evidence regarding the roles of PHC nurses remains limited, necessitating further clarification of their tasks and role functions in China.

## 2. Background

PHC serves as the “cornerstone” of health systems [13], focusing on health promotion, injury and disease prevention, and advancement of health equity and social well‐being. Currently, global aging and the increase in NCDs have emerged as critical public health worldwide, leading to increasing demand for basic health services and health promotion among populations [14]. PHC institutions are the optimal platform for delivering basic public health functions [15] and are regarded as the foundation and pathway for achieving UHC [1], playing a pivotal role in enhancing the physical and mental health and social well‐being of individuals across all ages throughout the life cycle [16].

Nurses, as frontline providers in PHC [17], play a crucial role in NCDs management, patient education, medication management, public health services, and healthcare administration [18, 19]. A quasiexperimental study in Spain demonstrated that health monitoring and health education for patients with complex chronic diseases significantly reduced hospital readmission rates and emergency department visits [20]. Moreover, the nurse‐led PHC model has been shown more effective in providing health‐related counseling to patients, leading to improved patient health outcomes and higher patient satisfaction [21]. A two‐arm pilot study showed that an intervention for T2DM patients based on the BCW model and developed and implemented by PHC nurses has been effective in improving adherence to healthy eating, exercise, and HbA1c levels [22]. Recognizing the vital role of PHC nurses, countries such as Australia, Canada, and New Zealand have established competencies standards and/or practice guidelines for PHC nurses, clearly defining role functions within PHC [23–25].

In China, the PHC system comprises urban community health service centers (CHSCs), community health service stations (CHSSs), township health centers (THCs), and village clinics [26]. Community health care encompasses a wide range of services, including disease prevention, health care, medical services, rehabilitation, health education, and family care planning guidance [27]. However, research has found that PHC nurses in China rarely engaged in services such as NCDs monitoring and management, health education, rehabilitation care, and end‐of‐life care [28, 29]. Additionally, studies by Li et al. [10] suggest that the roles of PHC nurses are often constrained by power imbalances, which negatively impact the quality of health services. In the past 5 years, the scope of tasks of PHC nurses has been expanded to include not only health promotion and management, and epidemiologic analysis of chronic and infectious diseases but also the rapid increase in the use of teleconsultation [30–32]. China has also introduced policies to improve public health capabilities, especially in the prevention, control, and treatment of major epidemics [33]. This has resulted in new tasks being delegated to PHC institutions, placing additional demands on the role functions of PHC nurses. However, it remains unclear whether PHC nurses can effectively fulfill these new tasks and meet the required role expectations. Ambiguity in role definitions can lead to unclear boundaries in nurses’ scope of practice, which may cause conflicts within interdisciplinary teams and result in suboptimal access to healthcare services for community residents [34].

Therefore, this study aims to investigate the tasks of PHC nurses in three Northeastern provinces of China in order to understand role functions to fully leverage the critical value of nurses in the PHC and meet the multilevel and diverse healthcare needs of community residents.

## 3. Methods

### 3.1. Design

This study was reported following the Good Reporting of a Mixed Methods Study (GRAMMS) framework [35] (Appendix A). Grounded in pragmatism [36], a mixed‐methods convergent study was adopted to explore the tasks and role functions of PHC nurses in Northeastern China, as quantitative research alone cannot fully elucidate their role functions. In this study, quantitative and qualitative data were conducted simultaneously and independently. Following data collection, the two datasets were analyzed separately, and the results were triangulated to integrate, interpret, and report the findings. The mixed‐methods approach allowed for the examination of nurses’ role functions from multiple perspectives and data sources. Triangulation of data from different sources enhanced the validity and credibility of the findings [37]. Qualitative data were used to interpret and contextualize the results of quantitative research, and quantitative data can also provide further insights into the qualitative findings.

### 3.2. Setting, Participants, and Sample

In this study, a subset of PHC institutions was randomly selected across three provinces in Northeast China, ensuring that each province included institutions from both urban and rural areas. For the quantitative survey, convenience sampling was used to recruit all eligible nurses from the selected institutions, who were invited to complete the self‐administered questionnaire. This approach ensured the inclusion of nurses with different professional titles. For the qualitative phase, electronic invitations were sent to recruit nurses from the same institutions via purposive sampling for focus group interviews. To avoid disrupting the normal operation of PHC institutions on the focus group interview day, each focus group consisted of at least six PHC nurses with different job responsibilities and professional titles, thus ensuring the representativeness of the sample. The same inclusion and exclusion criteria were applied to both the quantitative and qualitative studies. Inclusion criteria are as follows: (1) holding a valid nursing professional qualification certificate; (2) having worked continuously in a PHC institution for ≥ 2 years; (3) engaging primarily in clinical nursing or basic public health services; (4) providing informed consent and voluntarily participating in the study. Exclusion criteria are as follows: (1) student nurses, rotating nurses, or advanced training nurses; (2) nurses engaged in management or administrative work; (3) being absent from work during the survey period (e.g., annual leave).

### 3.3. Data Collection

Data collection was conducted from July to August 2024. For the quantitative study, paper‐based questionnaires were used to investigate nurses’ tasks. The study instrument consisted of two parts. The first part was a general information questionnaire, which included seven items regarding gender, age, initial educational degree, highest educational degree, years of practice, type of medical institution, and professional title. The second part was the Chinese Nurses’ Tasks Survey Questionnaire, which was revised and validated by the research team to better align with the current Chinese context. The revised questionnaire included 10 dimensions (e.g. routine and specialized nursing procedures, medication use and management, patient safety and occupational protection, collaboration and multidisciplinary cooperation, nursing activities to assist patients, patient monitoring and assessment, health education and health management, communication and psychological support, professional values, and other related tasks) with a total of 158 tasks, and the overall Cronbach’s *α* coefficient was 0.997. The evaluation criteria were execution frequency, importance, and familiarity (see Appendix B for a detailed description). However, since this study focused solely on the tasks performed by PHC nurses, only the execution frequency was used as the evaluation criterion. One researcher (Jialin Li) trained designated coordinators from the participating PHC institutions on questionnaire administration. Questionnaires were mailed to the coordinators, who distributed and subsequently collected the completed forms.

For the qualitative study, semistructured focus group interviews were adopted. Given that nurses may have considerable differences in their perceptions of their own roles, focus groups encouraged participants to engage in discussions and interactions to elicit diverse perspectives [38]. In addition, as PHC institutions are usually busy, focus groups were more efficient. Each focus group was conducted by three researchers (Jiashuo Zhang, Wenna Qian, and Jialin Li). The outline was developed based on a preliminary presurvey, covering the content of daily tasks as well as nurses’ perceptions, understandings, and attitudes toward relevant tasks. All focus groups were moderated by a researcher (Jiashuo Zhang) with rich experience in qualitative research, who was proficient in focus group moderation skills and had previous moderation experience. Another researcher (Wenna Qian) was responsible for audio recording and note‐taking. All focus group interviews were conducted in a quiet and private environment. Since the content involved nurses’ views on daily work, head nurses and hospital managers were requested to be absent. This measure was taken to alleviate nurses’ concerns, enable them to speak freely, and reduce bias in the results.

### 3.4. Data Analysis

Quantitative data were double‐entered into EpiData 3.1 software (Wenna Qian and Jialin Li) (EpiData Association, Denmark) by two independent researchers and cross‐validated for accuracy, and SPSS 27.0 software (IBM, Chicago, IL, USA) was used for quantitative data analysis (Jiashuo Zhang). Descriptive statistics were used to summarize the demographic characteristics of the participants, and mean and standard deviation were used to describe the execution frequency of tasks.

Natural language processing (NLP) is an important research area in artificial intelligence, enabling machines to understand, interpret, and generate human language. It is a valuable tool for extracting themes from documents. The BERTopic topic modeling method, a powerful tool for thematic analysis in the NLP, allows mapping documents from large‐scale textual datasets into topic space and automatically identifying latent topics. It utilizes the BERT model to capture semantic information from the documents and applies topic modeling techniques to cluster this semantic information, thereby extracting topics.

The audio recordings of the focus groups were transcribed verbatim into Microsoft Word documents within 24 h. First, Jieba (a Chinese word segmentation tool) was employed to segment the raw Chinese texts, followed by filtering out meaningless stop words based on a general Chinese stop‐word lexicon. Then, the BERTopic framework was configured with core parameters: the pretrained model BERT‐base‐Chinese was specified as the embedding_model to automatically generate text semantic vectors; meanwhile, the framework’s built‐in UMAP model was invoked to conduct dimensionality reduction on the vectors, and its built‐in HDBSCAN model was used to perform density‐based clustering. Ultimately, TF‐IDF was applied to extract the core topic words for each clustering cluster. Two researchers (Jiashuo Zhang and Jialin Li) conducted content analysis of the transcribed focus group data based on the clustered topics. The stability of the topics was verified by combining topic distribution maps, heatmaps, and keyword bar charts. If redundancies or outliers were identified, the model was optimized and retrained to ensure that the topics were both statistically significant and consistent with the real‐world context.

### 3.5. Integration

**FIGURE 1:**
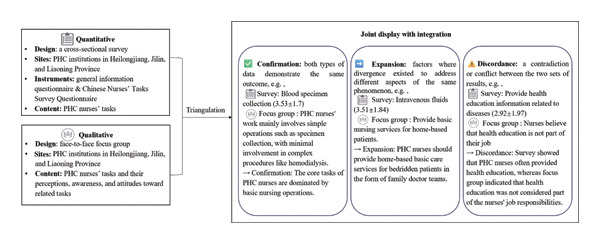
A diagram of this research.

Data triangulation was performed on quantitative and qualitative data, and a weaving approach was adopted to interpret and report the results (Jiashuo Zhang) [39]. First, the execution frequency of tasks from the quantitative results was juxtaposed with the qualitative themes, and the integrated findings were presented in narrative form. Three types of results were obtained: “confirmation” indicates that both types of data demonstrate the same outcome; “expansion” means that factors where divergence existed to address different aspects of the same phenomenon; “discordance” refers to a contradiction or conflict between the two sets of results [40] (see Figure 1 for the diagram of the research). Through joint discussion among three researchers, the integrated results were merged and refined, and the role functions of PHC nurses were finally identified.

### 3.6. Trustworthiness

During the content analysis of qualitative data, two researchers engaged in ongoing discussions and provided a detailed description of the results to enhance the credibility, dependability, and transferability of the findings [41]. When integrating the results, all researchers discussed and reached a consensus on the thematic categories and the integration results to further strengthen the credibility of the study.

### 3.7. Ethical Approval and Consent

This study was approved by the Ethics Committee of China Medical University (ethical approval no.: 2024No. 139). Prior to the initiation of the study, informed consent was obtained from all participants: Participants in the quantitative survey all completed written informed consent forms; in addition to completing written informed consent forms, participants in the qualitative focus group interviews also provided verbal informed consent, explicitly granting permission for audio recording during the research process.

## 4. Results

### 4.1. General Characteristics of Participants

A total of 123 valid questionnaires were collected from the quantitative survey conducted across three provinces (Table 1), with participants from urban PHC institutions (*N* = 65) and rural PHC institutions (*N* = 58). Specifically, the sample included 4 institutions from Jilin Province (*N* = 37), 4 from Heilongjiang Province (*N* = 61), and 2 from Liaoning Province (*N* = 25). The participants were predominantly nurses aged 20–40 years (63.4%), with the vast majority being females (97.6%). Most of them held a college diploma as their initial educational qualification (52.0%), and a large proportion possessed a primary professional title (55.3%).

**TABLE 1:** General characteristics of participants in quantitative surveys (*n* = 123).

Items	Categories	*N* (%)
Age	< 20	1 (0.8)
	20∼30	41 (33.3)
	30∼40	37 (30.1)
	40∼50	23 (18.7)
	≥ 50	21 (17.1)

Gender	Male	3 (2.4)
	Female	120 (97.6)

Initial education degree	Secondary vocational	39 (31.7)
	Associate degree	64 (52.0)
	Bachelor degree	20 (16.3)

Highest education degree	Secondary vocational	12 (9.8)
	Associate degree	45 (36.5)
	Bachelor degree	66 (53.7)

Years of practice	< 5	24 (19.5)
	5∼10	27 (22.0)
	10∼15	19 (15.4)
	15∼20	11 (8.9)
	20∼25	14 (11.4)
	≥ 25	24 (22.8)

Type of healthcare institution	Community health service centers	65 (52.8)
	Township health centers	58 (47.2)

Professional title	Junior	68 (55.3)
	Middle	55 (44.7)

Province	Jilin	37 (30.1)
	Heilongjiang	61 (49.6)
	Liaoning	25 (20.3)

Total		123

A total of 10 focus group discussions were conducted in three provinces for this study, including 4 sessions in Jilin Province (*N* = 33), 4 in Heilongjiang Province (*N* = 30), and 2 in Liaoning Province (*N* = 12). Each session involved 6–12 participants and lasted 20–41 min, with an average duration of 25.6 min. The general characteristics of the focus group interviews are presented in Table 2.

**TABLE 2:** General characteristics of participants for focus group interviews (*n* = 75).

No.	Site	Date	Number of participants (*N*)	Duration of focus group interviews (min)
	Jilin Province			
T1	Nanhu No. 1 Community Health Services Center	2024.07.24	6 (Junior 2, Middle 4) (Male 0, Female 6)	28
T2	Dongfang Community Health Services Center	2024.07.24	8 (Junior 3, Middle 5) (Male 1, Female 7)	20
T3	Yongchun Township Health Centers	2024.07.24	7 (Junior 3, Middle 4) (Male 0, Female 7)	31
T4	Leshan Township Health Centers	2024.07.24	12 (Junior 4, Middle 8) (Male 0, Female 12)	21

	Heilongjiang Province			
T5	Rongshi Community Health Services Center	2024.07.30	7 (Junior 3, Middle 4) (Male 0, Female 7)	20
T6	Liaoyuan Community Health Services Center	2024.07.30	6 (Junior 1, Middle 5) (Male 0, Female 6)	25
T7	Xinfa Township Health Centers	2024.07.31	10 (Junior 2, Middle 8) (Male 0, Female 10)	20
T8	Tuanjie Township Health Centers	2024.07.31	7 (Junior 2, Middle 5) (Male 1, Female 6)	41

	Liaoning Province			
T9	Fenghuangshan Community Health Services Center	2024.08.07	6 (Junior 2, Middle 4) (Male 0, Female 6)	23
T10	Yuantongpu Township Health Centers	2024.08.07	6 (Junior 2, Middle 4) (Male 0, Female 6)	27
Total	10		75	

### 4.2. Tasks and Role Functions of PHC Nurses

**FIGURE 2:**
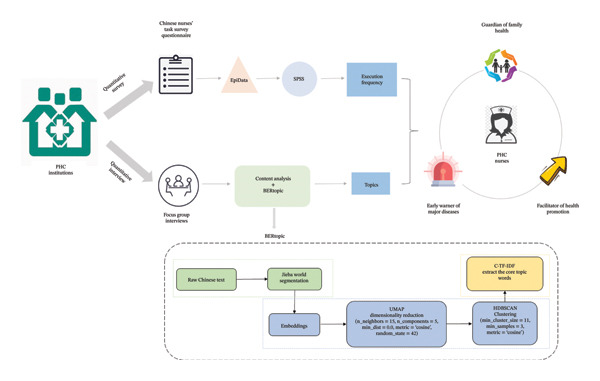
Overview of the research process. Description: This picture shows the whole process of this study. A mixed‐methods study was used to investigate the PHC nurses: the tasks of the nurses were investigated through questionnaires; the nurses’ perceptions, awareness, and attitudes toward the tasks were explored through face‐to‐face focus group interviews. Corresponding methods were used to analyze different data, and the two data were integrated to construct the role functions of PHC nurses. Three core role functions of Chinese PHC nurses were summarized and refined: guardian of family health (protect the health of all people), early warner of major diseases (monitor the health status of the public and provide warnings for major diseases.), and facilitator of health promotion (provide health education and guidance for the public).

The results indicated that the tasks of PHC nurses were mainly composed of providing basic medical services and conducting residents’ health management. They delivered comprehensive, full‐cycle health management services to patients and residents within the jurisdiction of PHC institutions (including CHSCs and THCs), aiming to maintain and promote individuals’ holistic health. By integrating the quantitative and qualitative results (see Table 3 for details), this study summarized and refined three role functions of PHC nurses, namely, guardian of family health, early warner of major diseases, and facilitator of health promotion (see Figure 2).

**TABLE 3:** Integration results of quantitative surveys and focus group interviews.

	Quantitative surveys		Focus group interviews	Integration
	(Item number) items	*M* ± SD		
Dimension 1. Routine and specialized nursing procedures	24. Blood specimen collection	3.53 ± 1.7	• PHC nurses’ work mainly involves simple operations such as specimen collection, with minimal involvement in complex procedures like hemodialysis;	• Confirmation: The core tasks of PHC nurses are dominated by basic nursing operations
	5. Intravenous fluids	3.51 ± 1.84	• Provide basic nursing services for home‐based patients	• Expansion: PHC nurses should provide home‐based basic care services for bedridden patients in the form of family doctor teams
	25. Urine specimen collection	3.14 ± 1.9		• Discordance: Nurses rarely carry out rehabilitation care, but home care services are actually medical resources to provide home rehabilitation for home‐based patients
	11. Intramuscular injection	3.06 ± 1.65		
	3. Intravenous injection	2.89 ± 1.87		
	9. Subcutaneous injection	2.8 ± 1.69		
	10. Intradermal injection	2.75 ± 1.72		
	36. Perform an electrocardiogram (ECG)	2.73 ± 1.79		
	76. Handle doctor’s orders	2.72 ± 2.15		
	77. Provide a conducive environment for treatment	2.68 ± 2.01		
	39. Perform the discharge process	2.64 ± 2.18		
	37. Perform the admission process	2.59 ± 2.19		
	1. Administration via the oral cavity (oral, sublingual, buccal, etc.)	2.49 ± 1.69		
	41. Perform oxygen therapy	2.43 ± 1.91		
	17. Inhalation administration (metered‐dose inhalers, aerosols, etc.)	2.42 ± 1.75		
	75. Record the treatment situation and patient response after the operation.	2.34 ± 1.98		
	50. Assist patients with activities of daily living (e.g. walking, repositioning, hygiene, eating, toileting)	2.2 ± 1.83		
	56. Perform a mouth wash	1 ± 1.39		
	61. Move the patient correctly	1.94 ± 1.75		
	51. Perform warm care	1.88 ± 1.74		
	6. The use of infusion pumps.	1.77 ± 1.68		
	52. Perform cooling care (e.g., using cooling blankets, ice packs)	1.75 ± 1.63		
	26. Stool specimen collection	1.68 ± 1.6		
	45. Catheter care	1.56 ± 1.58		
	33. Perform catheterization.	1.56 ± 1.56		
	72. Provide care tailored to the specific needs of elderly patients (activities, diet, etc.)	1.54 ± 1.67		
	54. Provide care to the inflamed or swollen areas and promote comfort (e.g., applying heat or cold therapy, elevating limbs, etc.)	1.53 ± 1.6		
	28. Throat swab specimen collection	1.45 ± 1.62		
	38. Perform the transfer process.	1.37 ± 1.42		
	42. Suction (oral, nasal, tracheal incisions)	1.22 ± 1.46		
	74. Maintain the continuity of care during the patient’s transfer to another hospital or department	1.2 ± 1.31		
	21. Bladder irrigation	1.12 ± 1.41		
	31. Perform an enema procedure	1.09 ± 1.45		
	16. Administer medication using ointment	1.06 ± 1.25		
	62. Nursing during surgery (arranging and protecting the sterile area, assessments during surgery, etc.)	1.03 ± 1.57		
	29. Insert a gastric tube	1.02 ± 1.41		
	46. Perform cardiopulmonary cerebral resuscitation (CPCR)	1.02 ± 1.09		
	2. Administration via the digestive tract (nasogastric tube, nasointestinal tube, etc.)	0.99 ± 1.17		
	63. Light therapy	0.98 ± 1.65		
	4. Venous transfusion	0.97 ± 1.4		
	57. Perform vulvar cleansing	0.94 ± 1.49		
	20. Intravesical instillation	0.92 ± 1.37		
	67. Postoperative care	0.88 ± 1.59		
	66. Preoperative preparation	0.86 ± 1.57		
	19. Administration via the rectum	0.85 ± 1.42		
	55. Application, maintenance, or removal of surgical instruments (e.g., splints, bandages)	0.85 ± 1.37		
	73. Provide end‐of‐life care to patients/families	0.85 ± 1.23		
	60. Perform gastric lavage	0.82 ± 1.2		
	43. Surgical wound drain care	0.81 ± 1.36		
	53. Wound/stoma care (e.g., wound irrigation, debridement care)	0.81 ± 1.28		
	15. Administer medication using an aerosol spray	0.78 ± 1.09		
	68. Remove the wound dressing and remove the stitches	0.76 ± 1.46		
	27. Sputum specimen collection	0.75 ± 1.24		
	34. Perform enteral nutrition (nasogastric feeding, gastrostomy, etc.)	0.72 ± 1.15		
	47. Heimlich maneuver	0.72 ± 1.04		
	18. Administration via the vagina	0.66 ± 1.34		
	35. Administering parenteral nutrition (such as TPN)	0.57 ± 1.08		
	32. Perform artificial airway care	0.57 ± 1.06		
	7. Administration via PICC	0.56 ± 1		
	44. Chest drain care	0.54 ± 1.07		
	69. Nursing during childbirth	0.53 ± 1.24		
	30. Insert nasogastric tube	0.53 ± 1.03		
	71. Monitoring fetal heart rate	0.51 ± 1.18		
	70. Postpartum care	0.5 ± 1.17		
	65. Neonatal care	0.49 ± 1.06		
	12. Administration by eye drops	0.49 ± 0.99		
	64. Prenatal care	0.47 ± 1.06		
	23. Administration via endotracheal intubation	0.45 ± 1.01		
	59. Perform eye wash	0.44 ± 0.93		
	13. Administration by ear drops	0.42 ± 0.94		
	58. Perform ear canal cleaning	0.42 ± 0.89		
	48. Use of ventilators	0.42 ± 0.83		
	14. Administration by nasal drops	0.41 ± 0.85		
	8. Administration via the port	0.39 ± 0.83		
	40. Performing corpse care	0.34 ± 0.88		
	49. Perform hemofiltration	0.26 ± 0.75		
	22. Ongoing peritoneal dialysis	0.26 ± 0.73		

Dimension 2. Medication use and management	79. Premedication assessment of the patient (e.g., vital signs, laboratory test results, history of allergies)	3.26 ± 1.83	• Provide medication care for patients;	• Confirmation: PHC nurses are required to provide medicine guidance for patients and residents
	83. Understand the relevant knowledge of drugs (drug effects, side effects, compatibility contraindications, etc.)	3.21 ± 1.85	• Conduct medication monitoring for patients with NCDs;	
	82. Observe and record the patient’s medication reaction during the medication process	3.16 ± 1.87	• Educate the vaccinated population on post‐vaccination precautions	
	80. Perform medication calculations (dose, concentration, speed, etc.)	2.95 ± 1.85		
	78. Evaluate the correctness of the doctor’s order (e.g., whether the medication is in line with the patient’s condition, whether the route of medication administration is appropriate, whether the dosage is appropriate)	2.92 ± 2.01		
	84. Custody and use of drugs (including narcotic and psychotropic drugs) in accordance with regulations	2.54 ± 1.93		
	81. Observation and maintenance of indwelling needles, PICCs, and infusion ports	1.78 ± 1.8		

Dimension 3. Patient safety and occupational protection	85. Perform the inspection system (e.g., operating room safety inspection)	3.86 ± 1.59	^∗^	• Expansion: Protecting patient safety and conducting occupational protection are principles that nurses must adhere to in their daily work
	96. Disposal of medical waste and other dangerous goods	3.7 ± 1.46		
	102. Correctly choose and wear occupational protective equipment	3.36 ± 1.5		
	95. Perform infection control measures (e.g., hand washing, isolation, disinfection and sterilization techniques, standard precautions)	3.28 ± 1.81		
	94. Proper implementation of shift handovers	3.07 ± 2.04		
	87. Perform an allergy test	2.94 ± 2.03		
	89. Ensure the proper and safe use of equipment in patient care and treatment procedures	2.9 ± 1.99		
	97. Perform emergency plans for medical institutions (including drills)	2.83 ± 1.52		
	101. Proper use of safety equipment (e.g. safety indwelling needles, safety lancets)	2.79 ± 1.77		
	93. Effective measures can be taken to prevent the occurrence of adverse events (e.g., falls, bed falls) during hospitalization	2.51 ± 2.04		
	90. Take measures to prevent/manage complications (e.g., circulatory complications, vertigo, asphyxia, potential neurological complications, allergies)	2.35 ± 1.98		
	91. Protect the integrity of the patient’s skin (e.g., Skin care, repositioning, use of pressure injury prevention pads)	2.29 ± 1.94		
	92. Correctly use restraints and safety facilities for patients as required by institutional regulations	2.26 ± 1.95		
	100. Properly perform occupational safety procedures (e.g., handling accidental injuries in chemotherapy, radiation, and nursing work)	1.54 ± 1.57		
	86. Correctly verify and transfuse blood products	1.37 ± 1.84		
	99. Report adverse events in the course of work (e.g., medical errors, patient falls, etc.)	1.37 ± 1.45		
	98. Report unsafe behaviors of other medical personnel (e.g., working under the influence of alcohol)	1.3 ± 1.67		
	88. Observe and record adverse reactions of blood products, and report as required	0.94 ± 1.53		

Dimension 4. Collaboration and multidisciplinary cooperation	106. Provide information to other medical personnel	2.54 ± 1.72	• The PHC nurse collaborates with general practitioners to form a family doctor team that provides home medical services, health education, and family doctor contract work;	• Confirmation: The multidisciplinary collaborative model of family doctor teams is the fundamental working form of PHC nurses
	105. Instruct other people (e.g. caregivers, cleaners)	2.48 ± 1.66	• Recommendations for consultations are provided to patients with fluctuating health levels	• Discordance: Nurses may perform tasks that do not typically belong to their responsibilities, such as debridement, in certain emergency situations
	107. Identify tasks/responsibilities that you cannot handle and seek assistance	2.41 ± 1.59	• When doctors are busy, nurses will also perform debridement for patients	
	104. Collaborate with other healthcare professionals to provide patient care (e.g., physicians, radiologists, nutritionists, laboratory staff)	1.9 ± 1.72		
	103. Cooperate with other healthcare professionals to complete procedures (e.g., catheterization, biopsy, debridement)	1.53 ± 1.8		
	108. Participate in multidisciplinary consultations and rounds	1.44 ± 1.47		

Dimension 5. Nursing activities to assist patients	115. Instruct the patient to follow medical advice for regular check‐ups	2.86 ± 1.92	• Provide medical, dietary, and exercise guidance for hospitalized patients, NCDs patients, and the elderly	• Confirmation: PHC nurses are required to provide patients with medical guidance and health lifestyle advice
	110. Assist patients in selecting a reasonable diet (e.g., helping with dieting, changing eating methods or frequency, arranging meals that align with ethnic characteristics)	2.8 ± 1.86		
	109. Assist patients in selecting appropriate activities	2.49 ± 1.7		
	114. Guide patients to correctly utilize community resources	2.23 ± 1.7		
	111. Assist patients and/or families in coping with unexpected issues after discharge	2.19 ± 1.78		
	113. Assist in rehabilitation exercises and treatment procedures.	1.61 ± 1.5		
	112. Assist patients in adapting to perceptual impairments (e.g., hearing, vision)	1.26 ± 1.32		

Dimension 6. Patient monitoring and assessment	116. Measure/evaluate the patient’s height and weight	3.59 ± 1.59	• Provide condition monitoring and assessment services for hospitalized patients;	• Confirmation: Conduct monitoring and evaluation of inpatients and patients with NCDs
	117. Monitor/evaluate the patient’s vital signs	3.46 ± 1.79	• Monitor the condition of patients with CNDs	
	119. Monitor/evaluate the patient’s blood glucose and blood lipids	3.33 ± 1.82		
	123. Conduct a general physical examination	3.24 ± 1.56		
	124. Collect and analyze health history to identify nursing issues	2.91 ± 1.69		
	126. Evaluate the effectiveness of the patient’s medication treatment	2.63 ± 1.86		
	118. Monitor/evaluate the patient’s water and electrolyte status (e.g., electrolytes, intake and output, edema, dehydration)	2.61 ± 1.91		
	128. Prioritization of patient care issues through assessment	2.54 ± 1.99		
	125. Evaluate the results of basic laboratory or auxiliary examinations	2.42 ± 1.8		
	129. Assess the patient’s pain level	2.37 ± 1.94		
	127. Perform risk assessments (e.g., visual impairment, potential falls, mobility classification, VTE risk assessment)	2.28 ± 2.01		
	120. Determine suicide risk	1.7 ± 1.72		
	122. Evaluate the family support system (e.g., structure, relationships, communication, boundaries, coping mechanisms)	1.55 ± 1.51		
	121. Assess the patient’s degree of drug/alcohol dependence, withdrawal, and intoxication	1.2 ± 1.43		

Dimension 7. Health education and health management	143. Establish a health record	3.09 ± 1.77	• Manage the health records of community residents;	• Confirmation: The establishment and management of health records is the task of PHC nurses; health education and NCDs management are important daily responsibilities of them
	133. Provide health education information related to diseases (such as smoking, safe sexual activities, etc.)	2.92 ± 1.97	• Nurses believe that health education is not part of their job;	• Expansion: Disease prevention is the duty of PHC nurses;
	132. Provide admission education and basic information about the disease to patients and/or their families	2.85 ± 2.02	• Provide health guidance and medical care advice to residents with NCDs;	• Discordance: Survey showed that PHC nurses often provided health education, whereas focus group indicated that health education was not considered part of the nurses’ job responsibilities
	137. Provide dietary guidance to patients and/or families (including types of food, eating positions, speed, temperature, etc.)	2.63 ± 1.93	• Popularize knowledge about healthy lifestyles, common NCDs, and infectious diseases to the entire audience;	
	140. Provide health care advice (such as regular check‐ups, immunizations, disease screenings, etc.)	2.63 ± 1.9	• Provide free health check‐up services for the elderly;	
	130. Provide medication guidance to patients and/or families (including methods, procedures, precautions, etc.)	2.59 ± 1.86	• Provide free screenings for the middle‐aged and elderly population for four high conditions (hypertension, high blood sugar, hyperlipidemia, high uric acid) and five cancers (lung cancer, liver cancer, colorectal cancer, female breast cancer, and cervical cancer);	
	141. Promote good interaction between patients and families	2.36 ± 1.76	• Implement health management for patients with hypertension and diabetes	
	135. Provide information to patients and/or families on how to safely use medical equipment	2.22 ± 1.69		
	136. Explain to patients and/or families about home safety knowledge	2.2 ± 1.63		
	142. Record the process and acceptance of education for patients and/or their families	2.03 ± 1.65		
	139. Instruct patients on how to manage pain	1.91 ± 1.73		
	131. Provide education to patients and/or families about growth and development information (e.g., growth and development, etc.)	1.8 ± 1.55		
	138. Provide education before and/or after surgery	1.05 ± 1.7		
	134. Provide perinatal education	0.86 ± 1.38		

Dimension 8. Communication and psychological support	144. Actively listen to the concerns of patients/families	2.95 ± 1.6	• There are very few people with mental illness in the community, such patients are in mental health centers. PHC institutions are only responsible for registering records	• Confirmation: PHC nurses engage less in psychological care and/or mental health care
	145. Help patients with emotional and spiritual needs	2.19 ± 1.45		
	148. Conduct psychological assessments for patients and provide psychological care for those with mental health issues or consult a psychologist	2.15 ± 1.69		
	147. Assess the psychological, social, spiritual, cultural, and occupational factors affecting the work of nursing patients	2.1 ± 1.51		
	146. Apply therapeutic communication skills to assist patients or families in understanding their own behaviors	2.07 ± 1.44		
	149. Inform families of patients with serious psychological problems that may lead to adverse outcomes (e.g., self‐harm or suicide) and take steps to enhance care	1.47 ± 1.34		

Dimension 9. Professional values	151. Protect patient privacy	3.93 ± 1.38	^∗^	• Expansion: The professional values are the guiding principles and norms rooted in the hearts of PHC nurses, permeating every aspect of their daily work
	152. Perform the notification system (such as informed consent)	3.85 ± 1.56		
	150. Respect the culture and beliefs of patients	2.93 ± 1.46		
	153. As a representative of patients (such as actively preventing actions that harm patients’ interests)	2.71 ± 1.74		

Dimension10. Other related tasks	155. Participate in on‐the‐job training	3.45 ± 1.4	• Participate in basic training such as first aid;	• Expansion: PHC nurses’ specialized training is insufficient; There is a lack of education for PHC nurses
	156. Participate in clinical nursing education activities (including teaching, lecturing, and guiding junior nurses/nursing students in operations, etc.)	2.39 ± 1.62	• Nurses have not studied PHC knowledge in school and learn from experienced nurses after entering the workplace	
	154. Participate in the quality control/improvement process (collecting data or participating in teams)	2.15 ± 1.84		
	158. Participate in or organize nursing administrative management activities	1.76 ± 1.78		
	157. Participate in or organize research activities related to nursing	1.59 ± 1.61		

^∗^The analysis results of the interview data do not include content related to this dimension.

#### 4.2.1. Guardian of Family Health

Focus group interviews and questionnaires revealed that the vast majority of PHC nurses did not perform disease treatment‐related nursing interventions. (T1) B4 (Narrator): “For example, subcutaneous injection, aseptic techniques during vaccination, and nebulization therapy are all basic routine nursing operations. We do not have access to complex procedures such as hemodialysis.” PHC institutions in China typically undertake basic public health services, including prevention and management of common diseases, fertility guidance, infectious disease prevention, disease screening, maternal and child health care, geriatric health care, rehabilitation guidance, referral services, and public health management. Nurses usually take on the primary responsibilities in these services, with a shift in the target population from a patient‐ and individual‐centered approach to a family‐ and healthy population‐centered approach. (T8) B4 (Narrator): “And like our job at the grassroots level, it’s just that the nature of the work is also more mixed. It’s that every nurse is multi‐skilled, we can’t just operate as a primary care nurse, but we also need to assist with public health.”

##### 4.2.1.1. Providing All‐Stage Basic Care Services for Homebound Patients

One of the key tasks of PHC nurses is to provide basic medical services to patients, particularly through home visits as part of a family doctor team that includes general practitioners. These services mainly involve basic nursing procedures, hospice care, and spiritual care that are not provided at present. A significant difference in the tasks between PHC nurses and general hospital nurses is the provision of home care. Nurses are required to work with physicians to provide home‐based basic medical or nursing care to bedridden patients, such as catheterization, nasal feeding, enemas, intramuscular injections, intravenous fluids, dressing changes, and suture removal. (T1) B3 (Narrator) “There are times when we catheterize patients who are bedridden and unable to move.”

##### 4.2.1.2. Providing Preventive Health Services for all Population

Disease prevention is a central component in all aspects of PHC. Nurses need to provide preventive vaccination (including planned and unplanned immunization), maternal and child health management, and health management services for middle‐aged and elderly people (e.g., free screenings for hypertension, hyperlipidemia, hyperglycemia, and hyperuricemia, as well as cancer screenings and health check‐ups for individuals aged 65 years and above). CHSCs and THCs take over all the work of child immunization programs, so a dedicated position for vaccination nurses has been set up to provide free vaccination services for children within the jurisdiction. In addition, vaccination has been extended to the entire population, including seasonal influenza vaccination for the elderly, HPV vaccination for women, and tetanus and rabies vaccination. As a result, PHC nurses are required to undertake a significant amount of vaccination service work. (T1) B3 (Narrator): “My part is vaccination, so children’s parents are closer to us, more like friends with us, and the children have to come (vaccinated) from zero to 6 years old.”

#### 4.2.2. Early Warner of Major Diseases

PHC nurses conduct health management and monitoring of major complications in patients with NCDs through the family doctor team model and provide timely medical advice or referral recommendations when patients’ conditions change. (T1) B5 (Narrator): “The residents come for a medical check‐up. During the physical examination, we ask him/her about blood pressure or blood sugar control, and if it is unusually high, we suggest that he/she come to the hospital for a consultation, or we ask him/her to be transferred to a higher‐level hospital for the next step of treatment.” The nurses in the focus group interviews mentioned NCDs management, focusing on the management of hypertension and diabetes. (T6) B2 (Narrator): “Chronic disease follow‐up, basically just hypertension and diabetes follow‐up.”

PHC nurses are required to visit homes to provide education on NCDs and cancer prevention. However, this process often presents challenges, particularly when dealing with elderly patients or those with poor compliance, which can hinder their ability to carry out their work effectively. Typically, PHC nurses are responsible for disease surveillance and early warning for individuals aged 65 years and above. However, physical limitations among the elderly can lead to resistance toward disease prevention guidance. (T8) B4 (Narrator) “There are older people who keep holding their glasses just because their eyes are blurred and they can’t read the small print.” There can also be a lack of trust faced, as in (T8) B4 (Narrator) “We have to tell him a lot, it’s a national program, how much it benefits you, before he can trust you. If you just give him a phone call, he doesn’t trust it.” (T1) B3 (Narrator): “So I think the high‐level hospital doctor he says it might be stronger and more authoritative.” However, the close proximity of PHC nurses to residents’ daily lives and the frequent exchange of health information create opportunities for building harmonious nurse–patient relationships. Professional guidance, supported by trust, is more likely to be accepted and followed by residents. (T8) B1 (Narrator): “Yeah, like we’re running this four‐high screening service right now, and so many of the residents come in because they know one of the doctors, one of the nurses, or one of the staff at our hospital.” Solid professional knowledge can also gain patients’ trust, (T8) B3 (Narrator): “The nurse’s knowledge base should be high, and you can coordinate better with the patient. He thinks that you, the nurse, have a good theoretical foundation and technical operation, then he recognizes you more.”

#### 4.2.3. Facilitator of Health Promotion

PHC also plays a critical role in health education targeted at patients attending PHC institutions and residents in the jurisdiction. For patients, nurses provide routine admission and discharge education, medication guidance, and so on. For residents, nurses and doctors formed a family healthcare team to provide health education. (T2) B6 (Narrator): “If there is a small point of the problem, such as hypertension/diabetes medication guidance, or blood sugar/blood pressure control is not good, and he (patient) wants to find someone to ask, then he (patient) can call the family doctor directly, and he (doctor) will give guidance over the phone without having to go out, which makes it very convenient. If it is found that there is any problem that cannot be solved by our PHC institutions, then our doctors will give the right guidance to tell patients what to do.” The content of health education includes the popularization of knowledge on common chronic and infectious diseases and guidance on healthy lifestyles. The health literacy of the residents is improved by organizing regular health science lectures and issuing health pamphlets. (T8) B1 (Narrator): “We are several ways to conduct health education, such as volunteer clinics or publicizing on World Health Day and Healthy Lifestyle Awareness Day.” Besides, nurses usually do not provide in‐depth personalized health education. (T2) B6 (Narrator): “If you ask questions, we nurses can roughly explain one or two things to you, but in‐depth if you want to understand in more detail, such as the medication piece, we follow the doctor’s order and listen to the doctor.” In addition, nurses also play an important role in the management of residents’ health records and the contracting of family doctors. (T5) B2 (Narrator): “We are in charge of the health records, and it’s quite perfect at the moment.”

However, focus group interviews revealed that not all PHC nurses agree that such tasks should fall within the scope of nursing responsibilities. (T8) B5 (Narrator) “I don’t think that evangelizing healthy people should be classified as something that nurses have to do. I don’t think it’s right or reasonable.”

## 5. Discussion

This study demonstrates that China is developing a family doctor team service model led by general practitioners and supported by PHC nurses. Team collaboration is a fundamental aspect of PHC nurses’ work, permeating their daily responsibilities. Within this model, nurse serves as guardian of family health, early warner of major diseases, and facilitator of health promotion. At present, the work of PHC nurses in China covers the entire range of national basic public health services [42]. PHC nurses’ workplaces are not only limited to PHC institutions but also include communities, township streets, and patients’ homes. Most of their work is conducted on health promotion days and during medical checkups, resulting in a time‐distributed pattern characterized by discontinuity and concentration.

Significant differences exist in the tasks of PHC nurses compared with those of nurses in general hospitals. The former provides basic nursing care for patients and preventive health services for the entire population, usually performing simple nursing procedures such as intravenous infusion and vaccination—these are also the most common tasks of PHC nurses [43]. Findings from a study on the work activities of PHC nurses in Poland demonstrated that their work still mainly consists of implementing physicians’ orders, with the most frequently performed tasks being intramuscular injection or intravenous infusion—results consistent with those of the present study [44]. It can thus be seen that the functions of PHC institutions (i.e., treating minor illnesses, managing chronic conditions, and delivering preventive care) determine the tasks of nurses. A review indicated that during the COVID‐19 pandemic, PHC nurses acted as comprehensive care providers for patients, who were required to conduct monitoring activities, pay close attention to patients with chronic diseases, and conduct home visits while fulfilling their daily tasks [45]. This functional connotation is consistent with the role of guardian of family health identified in this study. For home‐based patients, PHC nurses need to provide point‐to‐point care support. On the one hand, nurses deliver professional supportive nursing services and instruct patients on the proper use of nursing techniques, which can reduce the burden on family caregivers. On the other hand, home care services provided by family physician teams break down the barrier of a single patient care model, provide patients with sufficient medical resources for home rehabilitation, and deliver personalized and extended nursing services. Meanwhile, nurses organize and connect patients with the same disease within their jurisdiction to communicate with each other, creating an environment for patient peer education [46]. Community nurses are responsible for providing basic nursing support to home‐based patients after hospital discharge and assisting family caregivers in the postreferral rehabilitation of these patients. A cross‐sectional survey in Brazil also demonstrated that nurses are the core coordinators of continuous care for patients in PHC settings [47]. PHC nurses are the primary providers of home care, delivering continuous home‐based nursing services to patients with chronic diseases. However, the safety of home care cannot be guaranteed at present, posing potential risks to patient safety and the health and safety of medical staff themselves [48]. In addition, home care in China is still in the early stages of development, and the system of responsibilities and requirements has not yet been refined [49]. Furthermore, this study also found that there is a lack of communication and interaction between nurses and patients [50]. All these problems will prevent nurses from delivering high‐quality nursing services to patients [51]. Therefore, further attention should be paid to improving safety practices, and interventions to promote positive interaction should be developed and implemented, so that PHC nurses can better provide home care for home‐based patients and alleviate family care burdens.

With the advancement of population aging, service components such as management of common chronic diseases, prevention of major diseases, and geriatric health care have been gradually integrated into China’s PHC system. As early warner of major diseases, PHC nurses have undertaken more responsibilities in chronic disease management than before [29]. Their work covers a full spectrum of services, ranging from early disease prevention and control, medication management after diagnosis, to daily monitoring of disease progression, complication prevention, and lifestyle guidance. A study conducted in New Zealand demonstrated that lifestyle interventions for prediabetes led by PHC nurses could effectively reduce patients’ body weight and lower their risk of developing diabetes [22]. This has established PHC nurses as the first‐line of defense in PHC. However, in the course of delivering such services, nurses often encounter distrust from service recipients. This may stem from the entrenched stereotype in Chinese society that prioritizes physicians over nurses [52], and such distrust has hindered community nurses from providing services [53]. Nevertheless, the findings of this study indicated that close contact and effective communication with service recipients, as well as the improvement of nurses’ own professional competence, could mitigate such distrust and facilitate the delivery of nursing services, which is consistent with the research results of Kuo [30]. PHC nurses adjust nursing strategies based on their personal and professional experiences, values, efficiency, and clinical standards. Their roles extend beyond technical duties, positioning them not as passive implementers of clinical guidelines but as highly adaptable healthcare professionals [54]. In the meantime, in many countries (e.g., the United States), advanced practice nurses within the PHC system are granted prescription rights, equipped with professional autonomy, and accorded high occupational recognition [55], whereas such rights have not yet been conferred on nurses in China. Community nurses in China adjust their nursing strategies by balancing values, efficiency, and clinical standards based on personal and professional experience. Their role transcends simple technical duties: they are not passive implementers of clinical guidelines, but highly adaptable healthcare professionals [54]. Therefore, efforts should be made to create an enabling environment for PHC nurses to exercise nursing autonomy, thereby improving chronic disease management and public health outcomes.

The people‐centered, health‐focused tasks of PHC have led PHC nurses to take on extensive health education responsibilities. As facilitators of health promotion, nurses adopt diverse approaches to educate the public on common diseases and healthy lifestyles. However, this study found that the effectiveness of nurses’ health education remains suboptimal. Relevant research also indicated that PHC nurses in China needed to prioritize the development of competencies in health education and promotion [56]. This suboptimal effectiveness may be attributed to factors such as patients’ lack of interest in healthy lifestyles, insufficient time, or difficulties in modifying existing habits; additionally, nurses themselves often lack adequate knowledge regarding healthy lifestyle guidance [56].

Although PHC nurses in China have assumed the aforementioned roles, this study found that their role functions remain ambiguous and partially deficient. First, compared with mature PHC systems in countries such as the United Kingdom, China has notable gaps in areas such as palliative care and mental health nursing. This result was consistent with previous studies [28, 29]. The responsibilities of community nurses in the United Kingdom clearly include providing treatment, case management, disease management, rehabilitation support, disease prevention, health promotion and healthy behaviors, as well as palliative care and end‐of‐life care in community and home settings [57]. In addition, this study found that even when nurses were performing certain tasks, such as health education, they did not consider these tasks to be within the scope of nursing work. The possible reasons for this were as follows: first is insufficient education and training: the lack of PHC‐related education and inadequate on‐the‐job training led to unclear positioning of PHC nurses regarding their own functions. In most countries, the educational requirement for PHC nurses is a bachelor’s degree or diploma [58]. In contrast, the educational level of PHC nurses in China is generally low, most nurses have not studied PHC‐related courses during their school years, and after entering the workforce, PHC institutions have not provided effective on‐the‐job training, preventing them from accurately understanding their job responsibilities and tasks. Findings from an intervention study in Australia showed that orientation workshops, mentoring, and education activities could enhance the knowledge, skills, and confidence of nurses newly entering the PHC system [59]; second is shortage of human resources: low service remuneration, high work intensity, and stressful working environments for PHC nurses have led to a shortage of personnel [52, 53, 60], and unreasonable management has exacerbated this shortage. The workload, ethical pressure, and professional devaluation of PHC nurses have been continuously increasing [61]. An observational study in Slovenia indicated that PHC nurses usually switched between different activities every 3 minutes and handled multiple tasks simultaneously without adequate rest [62]. However, policies have entrusted more tasks to PHC institutions [63], resulting in a continuous increase in nurses’ workload. Many nurses hold multiple positions concurrently, leading to confusion about their own role functions. Third is imperfect policies and systems: Many medically developed countries have formulated comprehensive regulations for PHC nurses, clearly defining their legal rights and responsibilities in areas such as health promotion, disease management, and prescription rights [55, 64]. In particular, advanced practice nurses play a key role in improving the accessibility of medical services in areas with relatively limited physician resources. For example, Iceland has established diabetes outpatient nursing services and expanded the role of nurses to meet the needs of diabetic patients [55]. In contrast, China still lacks specialized national‐level regulations for PHC nurses, leaving nurses without a basis for their work and resulting in role deficiencies.

Therefore, managers should fundamentally clarify the role positioning and job responsibilities of nurses in PHC and leverage their advantages to improve the accessibility of PHC and medical services. First, corresponding policies and regulations should be formulated to clearly define the scope of work and legal boundaries of PHC nurses. Second, a training system for PHC nurses should be established; nurses should receive formal education and training before working in PHC institutions to better adapt to their roles and work. Finally, in terms of management, measures such as financial compensation and professional title promotion should be adopted to enhance the attractiveness of positions and alleviate the shortage of talents.

## 6. Strengths and Limitations

This study explored the tasks of PHC nurses in Northeast China from multiple perspectives using a mixed‐methods research approach, thereby identifying their role functions. The large sample size of the survey enhanced the credibility of the study findings. During the qualitative data analysis phase, the BERTopic model was applied to explore themes, which minimized researcher bias and rendered the findings more objective and reliable. However, this study also had certain limitations. First, the questionnaire used in the quantitative survey contained 158 items, which required a minimum of 20 min to complete. Nurses filled out the questionnaire during work breaks, which could lead to interruptions in the completion process. Meanwhile, the excessive number of items might cause questionnaire fatigue among nurses. Second, in the qualitative focus group interviews, although head nurses and hospital managers had been asked to avoid the sessions to maximize the likelihood of nurses expressing their true opinions, the interviewees included nurses at different hierarchical levels. Junior nurses might still experience pressure in the presence of senior colleagues, preventing them from freely expressing their views. In addition, this study was conducted in Northeast China and may not be representative of PHC nurses nationwide. Future studies should include nurses from different regions, especially economically developed areas in China, to enhance the generalizability of the results.

## 7. Conclusion

This study aimed to explore the current tasks and role functions of PHC nurses in China using a mixed‐methods research methodology. The results indicated that with the growing demand for national health care, the responsibilities of PHC nurses in China have expanded. They have undertaken all work tasks related to residents’ basic medical services and national basic public health services and assumed the roles of guardian of family health, early warner of major diseases, and facilitator of health promotion. However, the role functions of PHC nurses in China remain unclear at present and require further standardization.

## Author Contributions

Jiashuo Zhang: conceptualization; methodology; investigation; data curation; software; visualization; writing–original draft.

Wenna Qian: investigation; data curation; validation; formal analysis; writing–review and editing.

Jialin Li: writing–review and editing; supervision; project administration.

Mengyao Cui: methodology; formal analysis; project administration.

## Funding

This work was supported by the Health Human Resources Development Center of the National Health Commission of China [grant number: RCLX2314034].

## Disclosure

The views expressed in this study do not necessarily reflect those of the National Health Commission. The funder had no role in the study design, data collection, analysis and interpretation, writing of this paper, or decision to submit it for publication.

## Ethics Statement

Ethical approval for this study was obtained from the Ethics Committee of China Medical University (ethical approval no.: 2024No.139). Informed consent was obtained from all participants prior to the start of the study, with all participants completing an informed consent form, and verbal consent was obtained from qualitative interview participants, including permission for audio recording.

## Conflicts of Interest

The authors declare no conflicts of interest.

## Supporting Information

Additional supporting information can be found online in the Supporting Information section.

## Supporting information


**Supporting Information 1** Supporting Information A: A structured overview of the mixed methods design based on the GRAMMS framework.

**Supporting Information 2** Supporting Information B: Detailed definitions and scoring criteria for the three Likert‐scale dimensions assessed in the nursing task questionnaire.

## Data Availability

The raw data used to support the findings of this study have not been made available because participants were assured that raw data would remain confidential and not be shared.
