# Supplementary material for: Identifying Role Functions of Primary Health Care Nurses in China: A Mixed‐Methods Study in Three Northeastern Provinces
Source: J Nurs Manag. 2026 Mar 3;2026:3708836. doi: 10.1155/jonm/3708836 (PMC12954431; doi:10.1155/jonm/3708836)
Supplement: Supplementary file 1 — Supporting Information 1 Supporting Information A: A structured overview of the mixed methods design based on the GRAMMS framework. [file JONM-2026-3708836-s002.docx]

**Supplementary Material A: This study is based on the GRAMMS report**

A structured overview of the mixed methods design based on the GRAMMS framework.

| GRAMMS | corresponding manuscript |
| --- | --- |
| 1. Describe the justiﬁcation for using a mixed methods approach   to the research question | 3.1. Design (Page 8) |
| 1. Describe the design in terms of the purpose, priority and   sequence of methods | 3.1. Design (Page 8) |
| (3) Describe each method in terms of sampling, data collection  and analysis | 3.2. Setting, participants and sample (Page 8);  3.3. Data collection (Page 9);  3.4. Data analysis (Page 10) |
| (4) Describe where integration has occurred, how it has occurred  and who has participated in it | 3.5. Integration (Page 11) |
| (5) Describe any limitation of one method associated with the  present of the other method | 3.1. Design (Page 8);  6. Strengthens and limitations (Page 21) |
| (6) Describe any insights gained from mixing or integrating  methods | 3.5. Integration (Page 11) |
