# Supplementary material for: Identifying Role Functions of Primary Health Care Nurses in China: A Mixed‐Methods Study in Three Northeastern Provinces
Source: J Nurs Manag. 2026 Mar 3;2026:3708836. doi: 10.1155/jonm/3708836 (PMC12954431; doi:10.1155/jonm/3708836)
Supplement: Supplementary file 2 — Supporting Information 2 Supporting Information B: Detailed definitions and scoring criteria for the three Likert‐scale dimensions assessed in the nursing task questionnaire. [file JONM-2026-3708836-s001.docx]

**Supplementary Material B: Questionnaire Introduction**

Detailed definitions and scoring criteria for the three Likert-scale dimensions assessed in the nursing task questionnaire.

**Execution frequency** represents how many times you personally performed this task in the past month of work, using a 6-point Likert scale, with scores from 0 to 5 indicating “Never” (never performed this task in this ward), “Rarely” (performed this task less than 2 times in the past month), “Relatively little” (performed this task 2 or more times in the past month), “Average” (performed this task more than 2 times every week), “Relatively much “ (performed this task 1 time per day on average) and “Very much” (performed this task 2 or more times per day); **Importance** represents your assessment of the importance of this task, independent of the execution frequency, using a 4-point Likert scale, with a score of 1 to 4 indicating respectively “Not important” (this task can be completed at an appropriate time of your own choosing, and if not completed, it will not affect the quality of care), ‘Fair’ (this task is a task that should be completed, and if not completed, it will have a slight impact on the quality of care), “Comparatively important” (this task should be completed in a timely manner, and if not completed, it will have a noticeable impact on the quality of care) and ‘Very important’ (this task is of the highest priority for immediate completion, and failure or delay will have serious consequences affecting the patient's life); **Familiarity** represents the degree of mastery of the task as perceived by you during clinical nursing practice, using a 5-point Likert scale, with scores from 1 to 5 indicating “Understanding” (meaning that the task is known only at the level of knowledge and has not been implemented in clinical practice), “Familiar” (meaning that the task has been carried out in your clinical nursing practice, but you have not studied it in depth, and may encounter difficulties in practice and need to be guided by others to complete it), ‘Mastery’ (meaning that you can independently complete the task and accumulate experience in clinical practice), “Application” (meaning that you are able to perform the task independently and adapt it to the individual patient) and “Evaluation” (meaning that you are able to evaluate the task based on intrinsic evidence and external criteria).
